# Contribution of target alteration, protection and efflux pump in achieving high ciprofloxacin resistance in *Enterobacteriaceae*

**DOI:** 10.1186/s13568-016-0294-9

**Published:** 2016-12-21

**Authors:** Ram Prosad Chakrabarty, Munawar Sultana, Saadlee Shehreen, Selina Akter, M. Anwar Hossain

**Affiliations:** 1 Department of Microbiology, University of Dhaka, Dhaka, 1000 Bangladesh; 2 Department of Microbiology, Jagannath University, Dhaka, 1100 Bangladesh; 3 Department of Genetic Engineering and Biotechnology, University of Dhaka, Dhaka, 1000 Bangladesh; 4 Department of Microbiology, Jessore University of Science and Technology, Jessore, 7408 Bangladesh

**Keywords:** Ciprofloxacin resistance, DNA gyrase, Efflux pump, *Enterobacteriaceae*, *qnr*S

## Abstract

The study aims at revealing the comprehensive contribution of target alteration, target protection and efflux pump to the development of high level of ciprofloxacin (CIP) resistance in *Enterobacteriaceae* bacteria of environmental, clinical and poultry origins. Antibiotic susceptibility test was used to detect CIP resistant (CIPR) isolates and MIC_CIP_ was determined by broth microdilution method. The presence of *qnr*S gene was identified by PCR and Southern blot hybridization (SBH) confirmed their location. Checkerboard titration demonstrated the effect of NMP on CIP action. PCR followed by sequencing and in silico analysis revealed the contribution of mutations in *acr*R, *mar*R and *gyr*A to CIPR development. Out of 152 isolates, 101 were detected as CIPR. Randomly selected 53 isolates (MIC_CIP_ 4–512 µg/mL) were identified as *Escherichia* spp. (26), *Enterobacter* spp. (7), *Klebsiella* spp. (5) and *Salmonella* spp. (15) and of them 31 isolates carried *qnr*S. *qnr*S harboring 18 highly CIPR isolates (MIC_CIP_: 256–512 µg/mL) were selected for further study. SBH confirmed 7 isolates harbored *qnr*S gene in plasmids. The *acr*A, *acr*B and *tol*C were present in all 18 isolates and NMP had an additive (12-isolates) or synergistic (6-isolates) effect on CIP action. Most isolates contained double amino acid (aa) substitutions (S83L and D87N) in QRDR of GyrA resulting in an altered conformation of putative CIP binding pocket. However, some isolates contained single (S83L or S83Y) or no aa substitution but showed high CIPR implicating that the concerted action of three mechanisms is responsible for high CIPR with the most significant role of efflux pump.

**Electronic supplementary material:**

The online version of this article (doi:10.1186/s13568-016-0294-9) contains supplementary material, which is available to authorized users.

## Introduction

Ciprofloxacin (CIP) is a second generation fluoroquinolone and extensively used in the treatment of a wide range of infections caused by *Enterobacteriaceae*, and *Pseudomonas aeruginosa* (Kaplan et al. 2013; Oliphant and Green 2002). CIP usually exerts its effect by binding with targets such as DNA gyrase and DNA topoisomerase IV. However, frequent reports about the emergence of CIP resistance have created a conundrum regarding its use (Boyd et al. 2008; Lautenbach et al. 2004). So far, the emergence of resistance to CIP can be attributed to three known mechanisms such as protection of targets with Qnr protein, enhanced efflux pump expression and alteration in the quinolone-resistance determining-region (QRDR) of target enzymes (Alekshun and Levy 2007; Hooper 2001). Among these mechanisms, target alteration has been reported to be responsible for a high level of resistance to CIP whereas efflux pump and Qnr protein mediated mechanisms attributed to a low level of resistance (Jacoby 2005; Strahilevitz et al. 2009). Most of the previous studies focused on either single mechanism in many organisms or all three mechanisms in a single type (Kuo et al. 2009; Li et al. 2011; Lindgren et al. 2003; Tran and Jacoby 2002; Tran et al. 2005; Vanni et al. 2014). However, high resistance to this drug is emerging swiftly among *Enterobacteriaceae* leaving this drug ineffective against many infections and increasing the cost of treatment. Furthermore, insufficient comprehensive studies on CIP resistance mechanisms within highly resistant isolates would impede the attempts to increase the potency and decrease the resistance emergence by modifying the existing current drug or designing new one. Therefore, this investigation focused on addressing this fundamental gap in our knowledge by unveiling the contribution of different prevailing mechanisms to the development of high level of CIP resistance among multidrug resistant *Enterobacteriaceae* bacteria isolated from clinical waste water (CWW), urinary tract infection (UTI) and cloacal swabs of poultry (CSP) origins in Bangladesh for public health interest.

## Materials and methods

### Screening and selection of ciprofloxacin resistant *Enterobacteriaceae* isolates

A total of 152 presumptively identified MDR *Enterobacteriaceae* bacteria previously isolated from samples of 3 different origins such as CWW (24 isolates), UTI (61 isolates) and CSP (67 isolates) (Additional file 1: Table S1) were selected for initial screening of CIP resistance by the modified Kirby-Bauer disc diffusion method (Barry et al. 1985) and an organism was reported as susceptible, intermediate or resistant to CIP based on the diameter of zones of inhibition (Cockerill 2011).

### Identification of the *Enterobacteriaceae* isolates

All the isolates were identified on the basis of their growth phenotypes, Gram staining and biochemical properties according to the methods described in the “Manual of Methods for General Bacteriology (American Society for Microbiology (ASM) 1981)”. The biochemical results were used to identify the isolates presumptively using the tool BioCluster (Abdullah et al. 2015). The identification of the isolates was further verified by ARDRA (Amplified ribosomal DNA restriction analysis) grouping of 16S rRNA gene amplicons amplified using 27F and 1492R primers (Additional file 1: Table S2). The digestion was done using the *Alu*I (Promega, USA) restriction enzyme. The resulting digestion products were resolved by agarose gel electrophoresis using 1.5% agarose (w/v) gel running for 90 min at 70 V and the gel was viewed using Alpha Imager HP Gel-documentation system (Cell bioscience, USA). The restriction patterns were analyzed to cluster the genetically related isolates using the tool Phoretix 1D (Totallab, UK). The experimental controls used were uncut experimental DNA, digestion of commercially supplied control DNA and no-enzyme “mock” digestion. Two different size markers, 1 kb (Promega, USA) and 100 bp (Promega, USA) DNA ladders were used to analyze different restriction fragments. 16S rRNA gene amplicons of selected isolates representative of each genotype were sequenced followed by phylogenetic analysis to find out their close relatives (Nandi et al. 2013). The 16S rRNA gene sequences of the selected isolates have been deposited in the GenBank database (accession no. KT825916–KT825923). The GenBank accession numbers of previously identified isolates such as 26N, 28N, E36, E40, G3, G4 and 77 are KC542889.1, KC542890.1, KJ544200.1, KJ544201.1, KJ544205.1, KJ544206.1 and KF188422.1 respectively.

### Determination of MIC

The MIC of CIP (Wako Pure Chemical Industries Ltd, Japan) for the selected CIP-resistant *Enterobacteriaceae* isolates was determined by broth microdilution assay according to the Clinical and Laboratory Standards Institute (CLSI) guidelines (Wikler 2009). Microtiter plates were prepared by double dilution method so that each well of a 96 well microtiter plate contains 95 µL Mueller–Hinton Broth (MHB) and the concentration of the CIP ranges from 512 to 2 µg/mL. In each plate, two negative controls were used; one column contained MHB + 2 µg/mL ciprofloxacin (blank for the microtiter plate scanner) and another column contained MHB only (sterility control). All the wells in each row were inoculated with 5 µL (McFarland equivalent) of a particular organism except the negative controls. For each isolate, the MIC_CIP_ was determined in triplicate and the median MIC_CIP_ was recorded. The plate was incubated at 37 °C overnight at 300 rpm in a shaking incubator (WiseCube, Germany). When satisfactory growth was obtained (after 24–36 h) the plate was scanned with a microplate reader (Poweam Medical Systems Co., Limited, China) and the background OD was subtracted from the OD of each well. The bacterial cultures from the wells of microtiter plate were streaked on MHA containing 2 µg/mL ciprofloxacin to check the purity of the isolates.

### Screening of *qnr* gene within the *Enterobacteriaceae* isolates

Quinolone resistance encoding gene (*qnr*S) was investigated in selected isolates by PCR with a specific set of primers- *qnr*F and *qnr*R (Additional file 1: Table S2) and *qnr*S positive 18 isolates covering all genotypes with high MIC_CIP_ value (256–512 µg/mL) were selected for determining the location of *qnr*S gene by Southern blot hybridization. *qnr*S probe was prepared by PCR amplification and labeled with a PCR DIG-labeling kit (Roche Diagnostics GmbH, Mannheim, Germany) according to the instructions of the manufacturer. Plasmid DNA from the bacterial isolates and marker plasmids of *E. coli* V_517_ were extracted using Wizard Plus SV Minipreps plasmid DNA Purification kit (Promega, USA) and was separated in 0.8% agarose gel at 70 volts for 4 h. After depurination, denaturation, and neutralization of the gel, DNAs were transferred onto a Hybond N + nylone membrane (Nycomed Amershamplc, Buckinghamshire, UK) with a vacuum blotting system for 3–4 h and fixed by UV exposure. The membrane with blotted DNA was sequentially subjected to pre-hybridization and hybridization with a labeled probe. After hybridization, the DIG-High Prime DNA labeling and detection system (Digoxigenin Labeling and Detection Kit; Roche Diagnostics, Mannheim, Germany) was used for signal detection according to the manufacturer’s instruction.

### Efflux pump mediated ciprofloxacin resistance

The chromosomal DNAs extracted from the selected *Enterobacteriaceae* isolates were subjected to PCR using primers specific for *acr*A, *acr*B and *tol*C genes encoding AcrAB–TolC efflux pump complex and primers specific for efflux pump regulatory region genes *acr*R and *mar*R (Additional file 1: Table S2). Mutations within the regulatory proteins were studied after sequencing by bioinformatics analysis of the deduced amino acid sequences (Akter et al. 2012). PCR positive isolates were subjected to MIC_CIP_ determination by microdilution broth checkerboard technique in the presence and absence of an efflux pump inhibitor 1-(1-naphthylmethyl) piperazine (NMP) (SIGMA-ALDRICH, USA) in 96-well microtiter plates (Li et al. 2011). The checkerboard plates were inoculated with 10^5^–10^6^ CFU/mL each of bacterial culture and the final concentrations of NMP and CIP ranged from 512–4 µg/mL and 512–2 µg/mL respectively and bacterial growth was monitored after 24–36 h. The OD_600 nm_ of the plate was taken and the background OD was subtracted from the OD of each well. The interaction between the antibiotic and the inhibitor was interpreted on the basis of fractional inhibitory concentration (FIC) index where FIC indices of <0.5, 0.5 to <4.0 and >4.0 usually refer to synergism, additive and antagonism respectively (Braga et al. 2005; Li et al. 2011; Odds 2003).

### Analysis of mutation in *gyr*A gene

A 648 bp fragment of *gyr*A gene covering QRDR region (nucleotide position 199–318) of selected *Enterobacteriaceae* isolates (screened for Qnr and efflux pump) were amplified by PCR using primers *gyr*AF and *gyr*AR (Additional file 1: Table S2). The PCR amplicons were purified, sequenced and analyzed to find out amino acid substitutions. Reference amino acid sequences downloaded from NCBI (http://www.ncbi.nlm.nih.gov) (accession no. NP_416734.1, WP_047361088.1, WP_023280374.1, NP_461214.1) were compared with that of test isolates (accession no. KT825924-KT825939) and in silico site directed mutagenesis in a reference sequence (accession no. NP_416734.1) was carried out. Three dimensional (3D) homology models for both the reference and mutated sequences were built using SWISS-MODEL workspace (Arnold et al. 2006; Biasini et al. 2014). The best models determined by GMQE value and QMEAN values were obtained using the template 3lpx.1B which covered 56% of the query sequences with a sequence identity of 77.19 and 76.99% respectively for reference and mutated sequences. The energy minimization in YASARA (http://www.yasara.org/) refined this model and the Ramachandran plot was developed using Accelrys software package Discovery Studio Visualizer 2.0 (Studio 2013) to check whether the models were stereo-chemically favorable. The 3D models of GyrA homodimers were docked with a B form DNA (PDB ID: 1BNA) using ZDOCK 3.0.2 (Pierce et al. 2014) online server when arginine at position 47, histidines at position 78 and 80 and tyrosine at position 122, were selected as binding site on the DNA gyrase A homodimer for the DNA based on the information of active sites of DNA gyrase A subunit. The binary complex consisting of DNA gyrase subunit A and DNA was docked with ciprofloxacin (Drug Bank accession no. DB00537) using PatchDock web server (Duhovny et al. 2002) with clustering RMSD 1.5 (Akter et al. 2012).

## Results

### Ciprofloxacin resistance in *Enterobacteriaceae* isolates

**Fig. 1:**
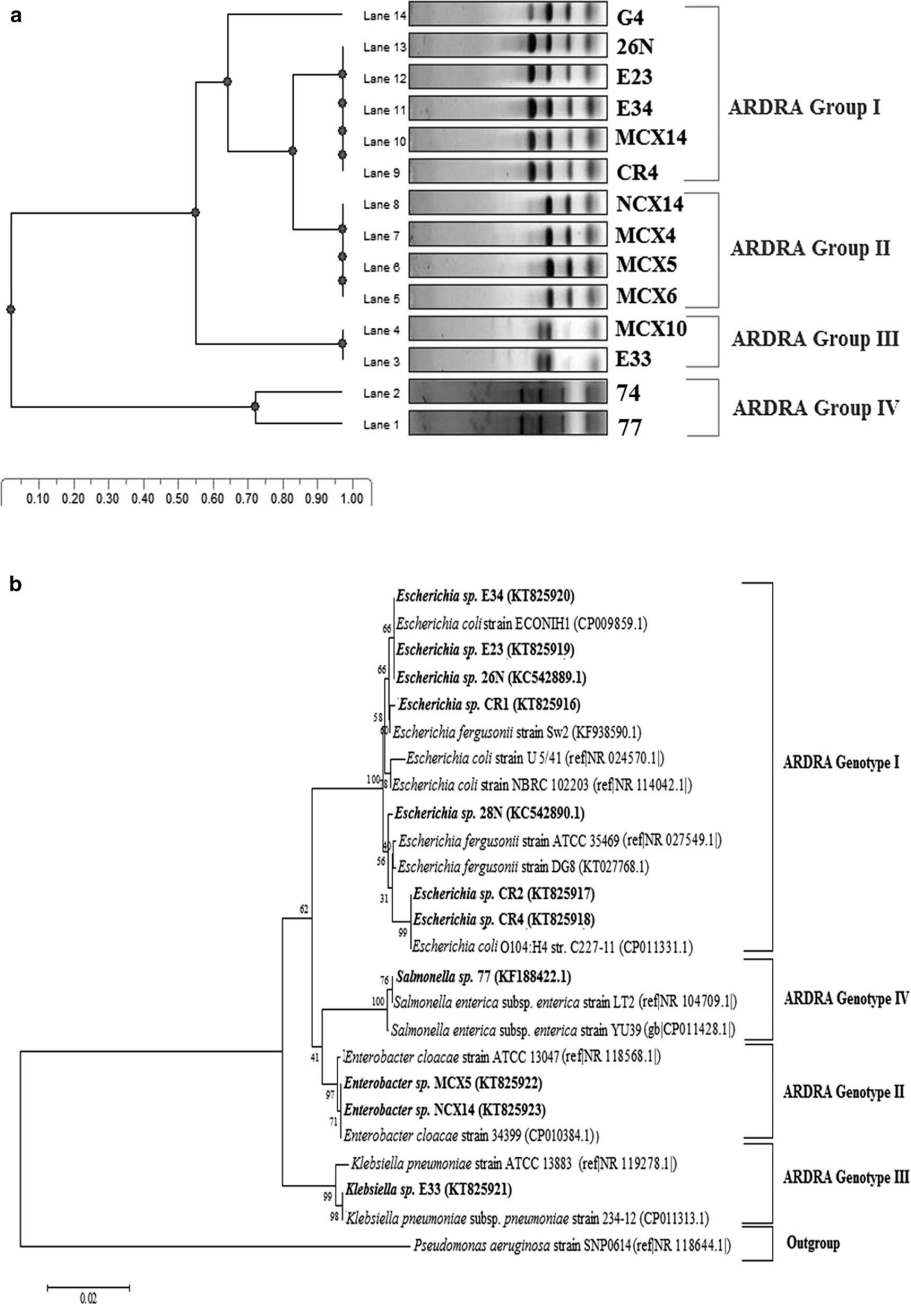
Phylogenetic analysis of isolates of Family *Enterobacteriaceae* using partial sequences of 16S rRNA gene. **a** Isolates were subjected to *Alu*I digestion followed by amplified ribosomal DNA restriction analysis (ARDRA) revealed 4 genotypic groups. **b** Phylogenetic tree constructed with MEGA6 based on ARDRA. The optimal tree was built using Neighbor-Joining method (sum of branch length = 0.2448)

Kirby-Bauer disk diffusion susceptibility test revealed that 101 out of 152 *Enterobacteriaceae* isolates were resistant to CIP in the order- CWW (~96%) > UTI (~72%) > CSP (51%) (Additional file 1: Table S1). Fifty-three *Enterobacteriaceae* isolates (23, CWW; 15, UTI; and 15 CSP) were selected for further study based on the growth and the biochemical properties, ARDRA grouping, 16S rRNA gene sequencing and phylogenetic analysis. All the analyses corroborated the results and revealed that the isolates representing ARDRA Group I, Group II, Group III and Group IV were closely related to *Escherichia* spp., *Enterobacter* spp., *Klebsiella* spp. and *Salmonella* spp. (Table 1; Fig. 1a, b).

**Table 1:** Identification of the isolates on the basis of conventional and molecular analysis

Isolate ID	Growth characteristics on different differential and selective media						Microscopic characteristics			
	Media	Appearance	Form	Elevation	Margin	Consistency	Gram-staining	Size	Shape	Arrangement
*28N*, *26N*, CR1, CR2, CR4, CR6, NCX9, MCX14, C6, C49, C79, C84, E8, E23, E29, E34, *E36*, E37, *E40*, E41, E42, E56, E58, G2, *G3*, *G4*	MAC	DP	C	F	E	D	Gram-negative	S	SR	Single
	EMB	BB, GMS								
	XLD	Y								
MCX1, MCX2, MCX3, MCX4, MCX5, MCX6, NCX4	MAC	P	C	R	E	G		M	Rd	
	EMB	B, DC								
	XLD	Y								
NCX6, MCX10, C1, C67, E33	MAC	LP	C	F	E	D		M	Rd	
	EMB	B, DC	C	F	E	G				
	XLD	Y	C	F	E	D				
18, 20, 36, 44, 45, 49, 54, 58, 60,68, 74, *77*, 81, 84, 94	MAC	Colorless	C	F	E	D		M	Rd	
	EMB	Color less	C	F	E	G				
	XLD	Red, BC	C	F	E	D				

Isolate ID	Biochemical characteristics													Presumptive organism	ARDRA genotype
	Sugar utilization tests			IMViC tests				Oxidase Test	KIA Test				Motility		
	Gl	Su	La	Indole	MR	VP	Citrate		Slant	Butt	Gas	H_2_S			
*28N*, *26N*, CR1, CR2, CR4, CR6, NCX9, MCX14, C6, C49, C79, C84, E8, E23, E29, E34, *E36*, E37, *E40*, E41, E42, E56, E58, G2, *G3*, *G4*	+	±	+	+	+	−	−	−	Y	Y	+	−	+	*Escherichia* spp.	I
MCX1, MCX2, MCX3, MCX4, MCX5, MCX6, NCX4	+	−	+	−	−	+	+	−	Y	Y	+	−	−	*Enterobacter* spp.	II
NCX6, MCX10, C1, C67, E33	+	−	+	−	−	+	+	−	Y	Y	+	−	−	*Klebsiella* spp.	III
18, 20, 36, 44, 45, 49, 54, 58, 60,68, 74, *77*, 81, 84, 94	+	−	−	−	+	−	+	−	Red	Y	−	+	−	*Salmonella* spp.	IV

Legends for conventional and molecular characteristics: *C* circular, *F* flat, *R* raised, *E* entire, *D* dry, *G* gummy, *S* short, *M* medium, *SR* short rod, *Rd* rod, *GMS* green metallic sheen, *DP* dark pink, *BB* blue black, *Y* yellow, *P* pink, *B* brown, *R* red, *DC* dark centered, *LP* light pink, *Gl* glucose, *Su* sucrose, *La* lactose, *BC* black centered

Italics isolates were previously identified in our laboratory

### MIC of ciprofloxacin and presence of *qnr*S gene within *Enterobacteriaceae* isolates

MICs of the ciprofloxacin (MIC_CIP_) for the selected 53 isolates were in the range of 4–512 µg/mL; among which 18 *Escherichia* spp., 4 *Enterobacter* spp., 5 *Klebsiella* spp. and 3 *Salmonella* spp. (3 out of 15 isolates) showed very high resistance to CIP (MIC_CIP_: 128-512 µg/mL) (Table 2). Among the 53 CIP resistant isolates, 31 possessed *qnr*S (a variant of *qnr* family; 19/26 *Escherichia* spp.; 7/7 *Enterobacter* spp.; 3/5 *Klebsiella* spp. and 2/15 *Salmonella* spp.) (Table 2). Based on high MIC values (256-512 µg/mL), 18 *qnr*S positive isolates from 4 different ARDRA groups were selected for exploring CIP resistance mechanisms within them (Table 2).

**Table 2:** Median minimum inhibitory concentrations (MICs) of ciprofloxacin for the 53 *Enterobacteriaceae* isolates and the presence of *qnr*S gene in the isolates

Isolates	Source	ID	*qnr*S	MIC (μg/mL)
*Escherichia* spp.	UTI	E8, *E23*, *E34*	+	512
		E29, E42	+	32
		E36, E37, E40	−	128
		E41, G3	+	128
		E56	+	8
		E58	+	4
		G2	+	128
		*G4*	+	256
	DMCH	*28N*	+	256
		*26N*	+	512
	SSMCH	*CR1*, *CR2*, *CR4*	+	512
		CR6	+	64
		*NCX9*, *MCX14*	+	256
		C6, C84	−	64
		C79	−	32
		C49	−	512
*Klebsiella* spp.	SSMCH	*NCX6*, *MCX10*	+	512
		*E33*	+	256
		C1	−	128
		C67	−	512
*Enterobacter* spp.	SSMCH	MCX4	+	4
		*MCX5*	+	512
		MCX2	+	32
		MCX3	+	8
		MCX1, *MCX6*, *NCX4*	+	256
*Salmonella* spp.	Poultry	36, 44, 45, 49, 54, 81, 84, 94, 60	−	16
		58, 68	−	32
		*74*	+	256
		*77*	+	512
		18	−	64
		20	−	128

Italics isolates were selected for revealing CIP resistance mechanisms

### Southern blot hybridization predicts *qnr*S gene location

**Fig. 2:**
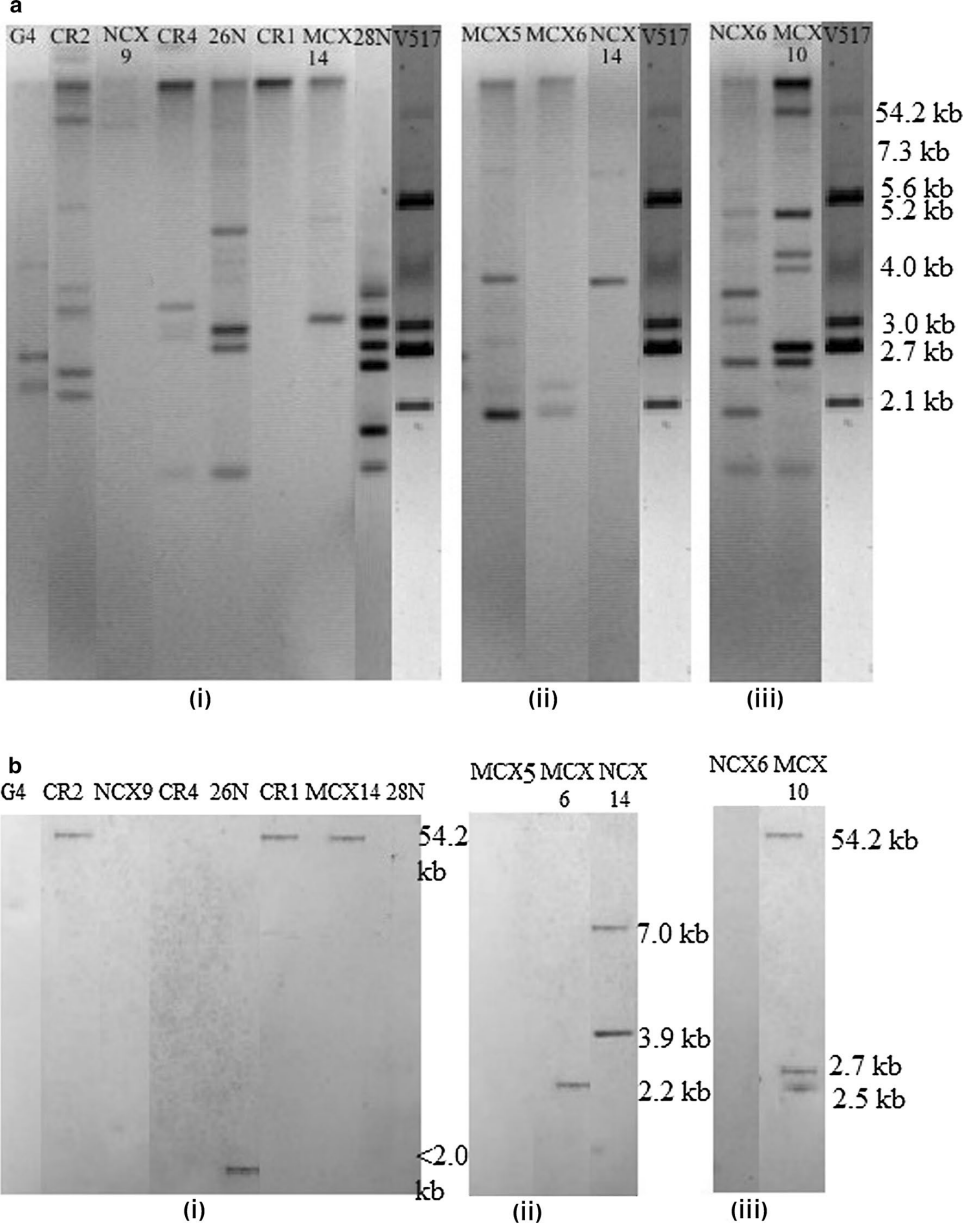
Confirming the location of *qrnS* gene in selected isolates. **a** Plasmid profiling of *Escherichia* spp. (*i*), *Enterobacter* spp. (*ii*) and *Klebsiella* spp. (*iii*) analyzed against V517 molecular mass markers followed by (**b**). Southern blot hybridization analysis for *qnr*S gene (*i*, *ii* and *iii* respectively) shows plasmids of different sizes containing *qnr*S gene

Out of selected 18 isolates, 13 isolates comprised of 8 *Escherichia* spp. (G4, CR2, NCX9, CR4, 26N, CR1, MCX14, and 28N), all 3 *Enterobacter* spp. (MCX5, MCX6 and NCX14) and 2 *Klebsiella* spp. (MCX10 and NCX6) harbored plasmids of different sizes (<2.0 to  >54.2 kb) although *Escherichia* *sp.* 28N and *Enterobacter* *sp.* NCX14 did not possess large plasmid >54.2 kb (Fig. 2; Table 3). Southern blot hybridization revealed that 3 *Escherichia* spp. (CR1, CR2 and MCX14) and *Klebsiella* *sp.* MCX 10 harbored the *qnr*S in the large plasmid (>54.2 kb). However, *Klebsiella* *sp.* MCX10 also showed positive result for two other plasmids of very small size (ca. 2.5 and 2.7 kb), which is of similar size to the *qnr*S harboring plasmid (2.2 kb) in *Enterobacter sp.* MCX6. *Enterobacter* *sp.* NCX14 was found to harbor the *qnr*S gene in two small plasmids of different sizes (ca. 3.9 and 7.0 kb). However, in *Escherichia* *sp.* 26N, a much smaller plasmid (<2.0 kb) was found to carry the *qnr*S gene (Fig. 2; Table 3).

**Table 3:** Overview of combined mechanism of ciprofloxacin resistance within *qnr*S, *acr*AB-*tol*C positive *Enterobacteriaceae*

Organism	ID	MIC (µg/mL)	Plasmid mediated *qnrS* gene		Efflux pump mediated resistance			Amino acid substitution in QRDR of DNA gyrase subunit A
			No. of plasmids extracted (approximate size in kb)	Size (in kb) of plasmid harboring *qnr*S gene	Effect of NMP on the action of CIP	Amino acid substitution in regulatory gene products		
						AcrR	MarR	
*Escherichia* spp.	E23	512	No plasmid	–	Additive	H115Y	G103S, Y137H, A53E	S83L, D87N
	E34	512	No plasmid	–	Additive	H115Y	G103S, Y137H, A53E	S83L, D87N
	26N	256	5 (<2.1, 2.7, 3.0, 4.6, >54.2)	1 (<2.0)	Additive	H115Y	G103S, Y137H, A53E	S83L, D87N
	28N	256	6 (<2.1, <2.1, 2.5, 2.8, 3.1, 3.9)	None	Additive	T213I, N214T	G103S, Y137H, K62R	S83L, D87N
	CR1	512	1 (>54.2)	1 (>54.2)	Synergistic	No substitution	G103S, Y137H	S83L
	CR2	512	7 (~2.3, 2.5, 3.7, 3.9, 5.2, 54.2, >54.2)	1 (>54.2)	Synergistic	No substitution	G103S, Y137H	S83L, D87N
	CR4	512	5 (<2.1, 2.9, 3.0, 3.5, >54.2)	None	Synergistic	ND	ND	S83L, D87N
	MCX14	256	2 (3.1, >54.2)	1 (>54.2)	Additive	ND	ND	S83L, D87N
	NCX9	256	2 (~54.2, >54.2)	None	Additive	No substitution	G103S, Y137H	No substitution
	G4	256	4 (~2.4, 2.7, 3.9, >54.2)	None	Additive	T213I, N214T	G103S, Y137H, K62R	S83L, D87N
*Enterobacter* spp.	MCX5	512	5 (2.0, 2.2, 2.8, 3.9, >54.2)	None	Synergistic	ND	ND	S83L, D87N
	MCX6	256	3 (2.0, 2.2, >54.2)	1 (2.2)	Additive	ND	ND	S83Y
	NCX14	256	1 (3.9, 7.0)	2 (3.9, 7.0)	Additive	ND	ND	S83L, D87N
*Klebsiella* spp.	E33	256	No plasmid	–	Additive	ND	ND	Not done
	NCX6	512	9 (<2.0, 2.0, 2.5, 3.0, 3.5, 4.8, 5.0, 54.2, >54.2)	None	Synergistic	ND	ND	Not done
	MCX10	512	8 (<2.0, 2.5, 2.7, 3.9, 4.0, 5.0, 54.2, >54.2)	3 (2.5, 2.7, >54.2)	Synergistic	ND	ND	S83L
*Salmonella* spp.	74	256	No plasmid	–	Additive	ND	ND	S83L, D87N
	77	512	No plasmid	–	Additive	ND	ND	S83L, D87N

*ND* not determined

### Contribution of efflux pump on resistance

All 18 isolates displayed positive PCR results for *acr*A, *acr*B and *tol*C genes encoding AcrAB-TolC efflux pump complex of Resistance-Nodulation-Division (RND) family (Table 3; Additional file 1: Figure S1). Checkerboard titration was employed to analyze the contribution of AcrAB-TolC efflux pump complex by determining the effect of NMP, an inhibitor of AcrAB-TolC efflux pump, on the action of CIP. The assay detected that in *Escherichia* spp. (CR1, CR2 and CR4), *Klebsiella* spp. (NCX6 and MCX10) and *Enterobacter* spp. (MCX5), NMP had a synergistic effect on the action of ciprofloxacin [FIC index (FICI) ≤ 0.5]. In all other isolates, NMP had an additive effect (0.5 < FICI ≤ 1) on the action of ciprofloxacin (Additional file 1: Table S3). Inhibition of AcrAB-TolC efflux pump significantly reduced the resistance to ciprofloxacin in selected 18 isolates. Furthermore, to detect the specific mutations in efflux pump regulatory genes, 8 *Escherichia* spp. (CR1, E34, G4, CR2, 28N, E23, 26N and NCX9) in some of which NMP had synergistic effect (e.g. CR1 and CR2) and in some of which NMP had additive effect (E34, G4, 28N, E23, 26N and NCX9) on the action of CIP were selected for amplification of *acr*R and *mar*R genes by PCR and sequencing (Table 3). Comparative analysis of amino acid sequences of *acr*R (accession no. KT825940-KT825947) and *mar*R (accession no. KT825948-KT825955) with that of references (accession no. NP_414997.1; accession no. NP_416047.4) revealed that in *acr*R gene, *Escherichia* spp. G4 and 28N contained the same double amino acid substitutions—T213I and N214T; and *Escherichia* spp. E34, E23 and 26 N contained the same single amino acid substitution- H115Y and in *mar*R gene, all isolates contained the same double amino acid substitutions (G103S and Y137H) (Table 3). In addition, *Escherichia* spp. 26N, E23 and E34 contained another amino acid substitution—A53E and *Escherichia* spp. G4 and 26N also contained another amino acid substitution—K62R in *mar*R gene (Table 3).

### Mutations in QRDR of *gyr*A

A 648 bp fragment of *gyr*A covering QRDR was targeted to amplify by PCR in 18 selected isolates. However, for 16 isolates, amplicon of desired size was obtained except *Klebsiella* spp. E33 and NCX6 (Table 3) and sequenced. The results revealed that all selected *Escherichia* spp. except NCX9 and CR1, two *Enterobacter* spp. (MCX5 and NCX14) and both *Salmonella* spp. 74 and 77 contained the same double amino acid substitutions (S83L and D87N). *Escherichia sp.* CR1 and *Klebsiella sp.* MCX10 contained the same single amino acid substitution (S83L) in the QRDR of *gyr*A which was S83Y for *Enterobacter* *sp.* MCX6. Interestingly, a highly CIP resistant *Escherichia* *sp.* NCX9 (MIC_CIP_ 256 µg/mL) did not contain any amino acid substitution in QRDR of GyrA subunit (Table 3).

## Discussion

Here, we report a very high and alarming level of CIP resistance in *Enterobacteriaceae* family of microorganisms, especially in opportunistic pathogens—*Escherichia* spp., emerging pathogens-*Enterobacter* spp., and well documented pathogens—*Klebsiella* spp. and *Salmonella* spp., belonging to clinical and poultry origin. Furthermore, this investigation conclusively demonstrated that all the 3-types of CIP resistance mechanisms—alteration of target enzyme, protection of target and efflux of the drug, were operative in *Enterobacteriaceae* isolates to attain the higher resistance. However, in contrast to our current knowledge that efflux pump is usually responsible for low level of CIP resistance (Hooper 2001; Jacoby 2005), this investigation demonstrated that efflux pump can contribute to a high resistance phenotype even in the absence of any mutation in the DNA gyrase subunit A.

### Abundance of ciprofloxacin resistance in MDR *Enterobacteriaceae* isolates

High level of resistance to CIP was found in isolates of CWW and UTI origins which seems cogent, because CIP has been widely used in the treatment of infections caused by both Gram-negative and Gram-positive microorganisms in the hospitals (Adnan et al. 2013; Kaplan et al. 2013). The presence of residual active fluoroquinolones in CWW exerts a selective pressure for the emergence, maintenance and horizontal transfer of resistant genes among microorganisms resulting in a complex resistant situation. The bacteria isolated from CSP also showed higher occurrence of CIP resistance, but MIC_CIP_ value was much lower than CWW and UTI isolates. This is probably due to low dosages of fluoroquinolone antibiotics used in poultry compared to human infection treatment. *Salmonella* spp. 74 and 77 of CSP origin were exceptional and could withstand very high concentration of CIP (MIC_CIP_ 256 and 512 µg/mL respectively) which insinuates a threat of the emergence of zoonotic infections. So far we know, there is no well-documented report of very high level of resistance (MIC_CIP_ 256–512 µg/mL) in *Enterobacter* spp., *Klebsiella* spp. and *Salmonella* spp. There are few reports available for *Escherichia* spp. with high MIC_CIP_ (128–256 µg/mL) (Sato et al. 2013a, b, c) but the molecular mechanisms underlying the ciprofloxacin resistance in them have not been explored in detail (Azmi et al. 2014; Lautenbach et al. 2010; Sato et al. 2013a, b, c; Weigel et al. 1998).

### Contribution of Qnr protein

A variant of *qnr* gene, *qnr*S, was found to be highly widespread within *Enterobacteriaceae* isolates of CWW and UTI origins. *Escherichia* spp. (19/26), *Enterobacter* spp. (7/7) and *Klebsiella* spp. (3/5) were PCR positive for *qnr*S gene. However, the occurrence of *qnr*S gene in *Salmonella* spp. (CSP origin) was very low (2/15). The *qnr*S negative isolates might contain other variants of *qnr* gene. Among the 18 selected *qnr*S positive isolates, 13 carried plasmids of different sizes (size ranged from <2.0 to >54.2 kb) (Table 3). Plasmid negative isolates *Escherichia* spp. E23, E34; *Klebsiella* *sp.* E33 and *Salmonella* spp. 74 and 77 might harbor chromosomal *qnr*S gene or have large plasmid that could not be retrieved in our experimental condition (Kuo et al. 2009).

Southern blot hybridization revealed that 7 out of 13 isolates harbored *qnr*S gene in the plasmids. Three *Escherichia* spp. isolates (CR1, CR2 and MCX14) along with *Klebsiella sp.* MCX10 carried *qnr*S gene in large plasmids of approximately same size (>54.2 kb) which is corroborated by the findings of other researchers (Kuo et al. 2009) but *Enterobacter* spp. NCX14 harbored the gene in small plasmids (3.9 and 7.0 kb) which could be two different plasmids or the same plasmids with different conformations. Although the presence of *qnr*S in small plasmid was unusual but not novel. Similar plasmids harboring *qnr*S was isolated from *Salmonella enterica* and *Aeromonas hydrophila* by other researchers (Hammerl et al. 2010; Han et al. 2012). However, *Escherichia* *sp.* 26N and *Enterobacter* *sp.* MCX6 were shown to carry *qnr*S in very small plasmids (<2.0 and 2.2 kb respectively) which was not reported earlier. In *Klebsiella* *sp.* MCX10, *qnr*S gene was carried in a large plasmid (>54.2 kb) along with two small plasmids (2.5 and 2.7 kb) which could also be the fragments of the large plasmid or could be acquired through vertical or horizontal transfer. Isolates from which no plasmid could be isolated or the isolates from which plasmids were isolated but did not hybridize with *qnr*S probe indicated that *qnr*S might be chromosome-borne. Alternatively, *qnr*S in these isolates could be borne by episomes, plasmids that can integrate with the chromosome which was reported by other researchers also (Kuo et al. 2009; Strahilevitz et al. 2009).

### Effects of efflux pump and its inhibitor on resistance

The selected *qnr*S positive and highly CIPR 18 isolates were equipped with active AcrAB-TolC efflux pump complex. Checkerboard titration revealed the synergistic effect of NMP on the action of CIP on three *Escherichia* spp. (CR1, CR2 and CR4, all of CWW origin), two *Klebsiella* spp., (NCX6 and MCX10) and *Enterobacter* *sp.* MCX5 (FICIs were <0.5) that means, the combined effect of NMP and CIP was higher than the sum of the individual effect, i.e. the efflux pump contributed more to the development of resistance to CIP than other two mechanisms. In remaining 13 isolates, NMP had an additive effect on the action of ciprofloxacin (FICIs were 0.5 < FICI ≤ 1.0) which means that efflux pump and mutations in QRDR and/or target protection by Qnr protein, all have significant role in the development of CIPR. Moreover, nonsynonymous mutations in efflux pump regulatory regions (*acr*R and *mar*R) of different *Escherichia* spp. indicate that the efflux pump expression might have been increased due to these mutations.

### Alteration of the QRDR of GyrA to the development of ciprofloxacin resistance

In this study, it was found that *Escherichia* spp. E23, E34, 26N, 28N, CR2, CR4, MCX14 and G4; *Enterobacter* spp. MCX5 and NCX14 and both *Salmonella* spp. 74 and 77 contained same double amino acid substitutions (S83L and D87N) in the QRDR of GyrA. The nucleotide change within a genus was also the same but within different genera, nucleotide change was different. S83L and D87N amino acid substitutions are reported to be associated with high level of CIPR (MIC_CIP_ ≥ 16 µg/mL) but not with such elevated level (MIC_CIP_ was 256–512 µg/mL) (Kaplan et al. 2013; Kocsis and Szabó 2013). However, highly CIPR *Escherichia* *sp.* CR1 (MIC_CIP_ 512 µg/mL), and *Klebsiella* spp. MCX10 (MIC_CIP_512 µg/mL) contained same single (S83L) amino acid substitution in QRDR of GyrA; and NMP had synergistic effect on the action of ciprofloxacin for them. In addition, highly CIPR *Enterobacter* *sp.* MCX6 contained single amino acid substitution (S83Y) whereas *Escherichia* *sp.* NCX 9 had no amino acid substitution in QRDR of GyrA but both isolates showed similar levels of resistance (CIP_MIC_ 256 µg/mL) and NMP had additive effect on the action of CIP for them. This means that even in the absence of mutation in QRDR, active efflux pump along with *qnr*S could contribute to high level of CIPR which differs from previous hypotheses that efflux pump and Qnr protein are only responsible for very low level of resistance (Hooper 2001; Jacoby 2005). According to our knowledge, there have been only two reports of fluoroquinolone resistant isolates (one was clinical isolate and another was laboratory derived strain) without any mutations in QRDR, however, they were just exceeding the breakpoint MIC of CIP (whereas MIC_CIP_ for *Escherichia* *sp.* NCX9. was 256 µg/mL) (Chopra and Galande 2011; Sato et al. 2013a, b, c). It was also observed in *Escherichia* spp. CR1, CR2 and CR4; *Enterobacter* *sp.* MCX5, *Klebsiella* spp. NCX6 and MCX10 that NMP had synergistic effect on the activity of CIP and exhibited the highest level of resistance (MIC_CIP_, 512 µg/mL) irrespective of single or double amino acid substitutions in QRDR of GyrA; although *Escherichia* *sp.* CR1, *Klebsiella sp.* MCX10 and *Enterobacter* *sp.* MCX6 could contain additional amino acid substitution in GyrB subunit or ParC subunit. However, isolates with double amino acid substitutions (S83L and D87N) and with additive effect of NMP on CIP action showed very high level of resistance (MIC_CIP_ 256–512 µg/mL). So it can be inferred that in case of selected *Enterobacteriaceae* isolates high level of CIPR resulted from the cumulative action of all three mechanisms of resistance to CIP with the mandatory requirement of active efflux pump.

**Fig. 3:**
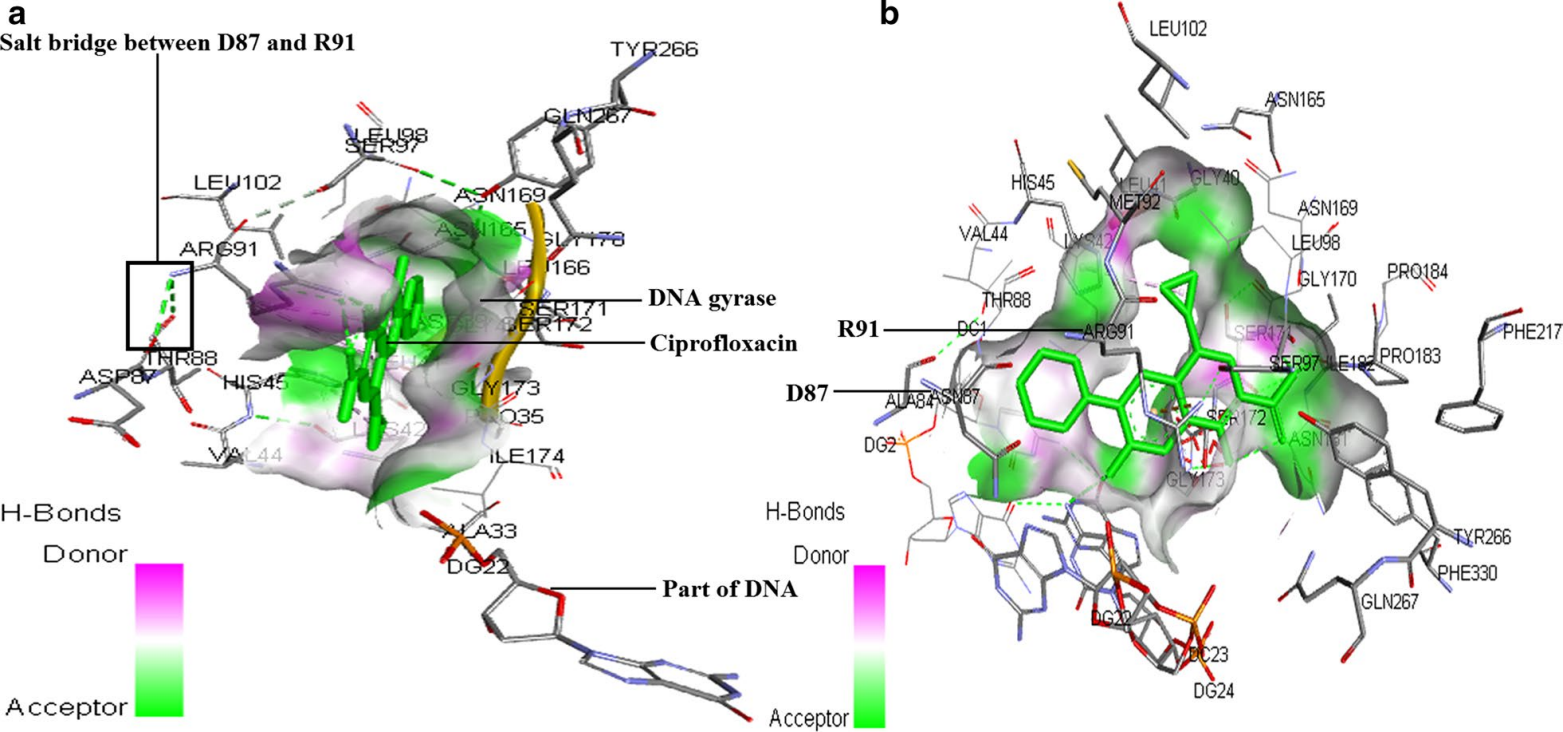
Interaction of ciprofloxacin in ciprofloxacin-DNA gyrase-DNA ternary complexes. The conformation of putative ciprofloxacin binding pocket appeared to alter in mutant gyrase A (**b**) as compared with the reference wild type gyrase A (**a**)

Although the nucleotide variation in QRDR between different species was up to 14.1%, but in comparison to the *E. coli* str. K-12 substr. MG1665 (NC_000913.3), the variation was mostly 4.4% (Additional file 1: Table S4) and therefore structural analysis was performed based on the amino acid sequence of GyrA of this organism (accession no. NP_416734.1) which is sensitive to CIP and an amino acid sequence derived from this sequence by in silico site directed mutagenesis with two amino acid substitutions—S83L and D87N (most common type of amino acid substitutions found in this study). Based on homology modelling and protein–ligand docking to produce ternary ciprofloxacin-GyrA-DNA complex, it was found that QRDR of GyrA constitutes the quinolone binding pocket and amino acids alteration can diminish the affinity of quinolone binding. It was elucidated that D87N mutation disrupt the salt-bridge formation between D87 and R91 resulting the change in drug binding pocket conformation. But the role of mutation at 83 position which occurred in almost all CIP *Enterobacteriaceae* isolates and also abundantly reported in literature has not been clear from in silico analysis. Therefore, further analysis to elucidate the role of 83-position mutation is needed for understanding the fluoroquinolone resistance mechanism (Fig. 3).

The present study conclusively demonstrated that *Enterobacteriaceae* isolates of different sources is being resistant to a very high and clinically significant concentration of ciprofloxacin (MIC ~ 512 µg/mL) by acquiring multiple resistance mechanisms in Bangladesh which has not been previously reported. Furthermore, in contrast to earlier reports, it was observed that efflux pump played a major role in introducing high level of ciprofloxacin resistance in the *Enterobacteriaceae* isolates, although concerted activity of all three reported mechanisms of fluoroquinolone resistance such as efflux pump, amino acid substitution in DNA gyrase A and Qnr were operative in most of the isolates.

## Additional file

**Additional file 1.** Additional tables and figure.

## Abbreviations

CIP: ciprofloxacin
CIPR: ciprofloxacin resistant
MIC: minimum inhibitory concentration
SBH: Southern blot hybridization
NMP: 1-(1-naphthylmethyl) piperazine
PCR: polymerase chain reaction
QRDR: quinolone resistance determining region
CWW: clinical waste water
UTI: urinary tract infection
CSP: cloacal swabs of poultry
MDR: multidrug resistant
OD: optical density
FIC: fractional inhibitory concentration
FICI: FIC index
ARDRA: amplified ribosomal DNA restriction analysis
RND: resistance-nodulation-division
GyrA: DNA gyrase subunit A
GyrB: DNA gyrase subunit B
