# Supplementary material for: Contribution of target alteration, protection and efflux pump in achieving high ciprofloxacin resistance in Enterobacteriaceae
Source: AMB Express. 2016 Dec 21;6:126. doi: 10.1186/s13568-016-0294-9 (PMC5177599; doi:10.1186/s13568-016-0294-9)
Supplement: Supplementary file 1 — Additional file 1. Additional tables and figure. [file 13568_2016_294_MOESM1_ESM.docx]

Table S1: *Enterobacteriaceae* isolates selected for study and occurrence of ciprofloxacin resistance in selected *Enterobacteriaceae* isolates

| Source (No of isolates) | Isolate ID | Ciprofloxacin Resistant Isolates (% of resistance) | References |
| --- | --- | --- | --- |
| Clinical Waste Water (DMCH & SSMCH) (24) | 28N, 26N, CR1, CR6, CR2, NCX9, MCX1, C84, C79, C6, C49, CR4, NCX6, MCX10, C1, C67, MCX4, MCX5, MCX2, MCX3, MCX6, MCX1, NCX4, C47 | 28N, 26N, CR1, CR6, CR2, NCX9, MCX14, C84, C79, C6, C49, CR4, NCX6, MCX10, C1, C67, MCX4, MCX5, MCX2, MCX3, MCX6, MCX1, NCX4 (95.83 %) | (Adnan, Sultana, Islam, Nandi, & Hossain, 2013) and laboratory stock |
| Urine samples of UTI patients (61) | E1, E4, E5, E6, E7, E8, E10, E11, E12, E13, E14, E16, E17, E18, E19, E20, E23, E28, E29, E30, E31, E32, E33, E34, E35, E36, E37, E38, E39, E40, E41, E42, E43, E45, E46, E47, E48, E51, E53, E55, E56, E58, E59, E61, E62, E64, E67, E69, E71, E74, E78, E79, E80, E81, E82, E83, E84, G1, G2, G3, G4 | E1, E8, E11, E12, E14, E16, E17, E18, E19, E20, E23, E28, E29, E30, E31, E32, E33, E34, E36, E37, E39, E40, E41, E42, E43, E45, E47, E48, E53, E56, E58, E59, E60, E64, E69, E74, E78, E80, E81, E83, G1, G2, G3, G4 (72.13%) | Laboratory stock |
| Cloacal swabs of poultry (67) | 10, 13, 41, 42, 43, 18, 19, 20, 60, 61, 62, 63, 64, 65, 74, 75, 76, 77, 79, 80, 81, 82, 83, 84, 88, 90, 91, 92, 93, 94, 95, 97, 100, 102, 44, 45, 46, 47, 48, 55, 56, 4, 5, 7, 9, 25, 33, 34, 36, 49, 50, 57, 58, 59, 66, 68, 70, 72, 103, 29, 30, 31, 32, 51, 52, 53, 54 | 18, 20, 36, 42, 44, 45, 48, 49, 51, 53, 54, 56, 58, 59, 61, 63, 64, 65, 66, 68, 74, 75, 76, 77, 80, 81, 83, 84,88, 90, 92, 93, 94, 60(50.75%) | (Nandi, Sultana, & Hossain, 2013; Sultana, Bilkis, Diba, & A. Hossain, 2014) and laboratory stock |

DMCH= Dhaka Medical College Hospital, SSMCH= Sir Salimullah Medical College Hospital, UTI= Urinary Tract Infection

*Bold isolates were selected for further study.

Table S2: Primer sequences used for the amplification of desired genes by PCR and corresponding annealing temperature.

| Target Gene | Primers | Sequences (5’ to 3’) | Amplicon size (bp) | Annealing temperature (^o^C) | Reference |
| --- | --- | --- | --- | --- | --- |
| 16S | 27F | 5’-AGAGTTTGATCCTGGCTCAG-3’ | 1430 | 55^o^C | (Lane, 1991) |
|  | 1492R | 5’-TACGGTTACCTTGTTACGACTT-3’ |  |  |  |
| *qnr*S | qnrF | 5’-GACGTGCTAACTGCGTGAT-3’ | 388 | 58 ^o^C | (Bouchakour et al., 2010) |
|  | qnrR | 5’-AACACCTCGACTTAAGTCTGA-3’ |  |  |  |
| *gyr*A | gyrA F | 5’-TACACCGGTCAACATTGAGG-3’ | 648 | 64 ^o^C | (Martínez-Martínez, Pascual, García, Tran, & Jacoby, 2003) |
|  | gyrA R | 5’-TTAATGATTGCCGCCGTCGG-3’ |  |  |  |
| *acr*A | acrA F | 5’-CTCTCAGGCAGCTTAGCCCTAA-3’ | 106 | 56 ^o^C | (Swick, Morgan-Linnell, Carlson, & Zechiedrich, 2011) |
|  | acrA R | 5’-AACAGTCAAAACTGAACCTCTGCA-3’ |  |  |  |
| *acr*B | acrB F | 5’-GGTCGATTCCGTTCTCCGTTA-3’ | 104 | 55 ^o^C | (Swick et al., 2011) |
|  | acrB R | 5’-ATGACGTTTACTTCCAGGTAG-3’ |  |  |  |
| *tol*C | tolC F | 5’-AAGCCGAAAAACGCAACCT-3’ | 100 | 54 ^o^C | (Swick et al., 2011) |
|  | tolC R | 5’-GATGGTCACTTACCGACTCTG-3’ |  |  |  |
| *acr*R | AcrR  8900F | 5’-ACTGTTACTACGCCAACG-3’ | 1000 | 51 ^o^C | (Lindgren, Karlsson, & Hughes, 2003) |
|  | AcrR  9934R | 5’-CTGAACCTGAAGAACGACCTG-3’ |  |  |  |
| *mar*R | MarORF1139 | 5’-GCCAGGCCAAGAAATAACGC-3’ | 840 | 56 ^o^C | (Lindgren et al., 2003) |
|  | MarORR2011 | 5’-GAGTAACCCGAACGCTCTGA-3’ |  |  |  |

Table S3: Effect of NMP on the action of ciprofloxacin as seen from significant reduction of MIC; an FICI value ≤ 0.5 indicates synergistic effect and 0.5<FICI≤1 indicates additive effect.

| Organism | Isolate ID | Median MIC (cip only) (µg/mL) | Median MIC (NMP only) (µg/mL) | /Median MIC (cip combined) (µg/mL) | Median MIC (NMP combined) (µg/mL) | FICI | Comment |
| --- | --- | --- | --- | --- | --- | --- | --- |
| *Escherichia* spp. | G4 | 256 | 128 | 4 | 64 | 0.515625 | Additive |
|  | CR1 | 512 | 256 | 64 | 32 | 0.25 | Synergistic |
|  | MCX14 | 256 | 256 | 128 | 128 | 1 | Additive |
|  | E23 | 512 | 128 | 256 | 16 | 0.625 | Additive |
|  | E34 | 512 | 256 | 128 | 128 | 0.75 | Additive |
|  | CR4 | 512 | 256 | 64 | 64 | 0.375 | Synergistic |
|  | CR2 | 512 | 256 | 128 | 4 | 0.265625 | Synergistic |
|  | 26N | 512 | 256 | 128 | 128 | 0.75 | Additive |
|  | 28N | 256 | 256 | 128 | 128 | 1 | Additive |
|  | NCX9 | 256 | 128 | 128 | 32 | 0.75 | Additive |
| *Enterobacter* spp. | NCX14 | 256 | 256 | 128 | 64 | 0.75 | Additive |
|  | MCX5 | 512 | 256 | 128 | 16 | 0.3125 | Synergistic |
|  | MCX6 | 256 | 128 | 128 | 64 | 1 | Additive |
| *Klebsiella* spp. | E33 | 256 | 128 | 128 | 16 | 0.625 | Additive |
|  | NCX6 | 512 | 256 | 128 | 64 | 0.5 | Synergistic |
|  | MCX10 | 512 | 256 | 128 | 32 | 0.375 | Synergistic |
| *Salmonella* spp. | 74 | 256 | 256 | 128 | 64 | 0.75 | Additive |
|  | 77 | 512 | 256 | 128 | 128 | 0.75 | Additive |

Table S4: Pairwise similarity between intra and inter species QRDRs of *gyr*A of selected isolates.

| Species | % Identity | | | | | | | | | | | | | | | | | | | |
| --- | --- | --- | --- | --- | --- | --- | --- | --- | --- | --- | --- | --- | --- | --- | --- | --- | --- | --- | --- | --- |
|  | A | B | C | D | E | F | G | H | I | J | K | L | M | N | O | P | Q | R | S | T |
| A |  | 95.6 | 95.6 | 96.5 | 95.6 | 95.6 | 95.6 | 95.6 | 95.6 | 95.6 | 100 | 91 | 95.6 | 90 | 95.6 | 86.9 | 96.5 | 88.9 | 95.6 | 95.6 |
| B |  |  | 100 | 99.2 | 100 | 100 | 100 | 100 | 100 | 100 | 95.6 | 91 | 100 | 91 | 100 | 86.9 | 99.2 | 88.9 | 100 | 100 |
| C |  |  |  | 99.2 | 100 | 100 | 100 | 100 | 100 | 100 | 95.6 | 91 | 100 | 91 | 100 | 86.9 | 99.2 | 88.9 | 100 | 100 |
| D |  |  |  |  | 99.2 | 99.2 | 99.2 | 99.2 | 99.2 | 99.2 | 96.5 | 92 | 99.2 | 92 | 99.2 | 87.9 | 100 | 89.9 | 99.2 | 99.2 |
| E |  |  |  |  |  | 100 | 100 | 100 | 100 | 100 | 95.6 | 91 | 100 | 91 | 100 | 86.9 | 99.2 | 88.9 | 100 | 100 |
| F |  |  |  |  |  |  | 100 | 100 | 100 | 100 | 95.6 | 91 | 100 | 91 | 100 | 86.9 | 99.2 | 88.9 | 100 | 100 |
| G |  |  |  |  |  |  |  | 100 | 100 | 100 | 95.6 | 91 | 100 | 91 | 100 | 86.9 | 99.2 | 88.9 | 100 | 100 |
| H |  |  |  |  |  |  |  |  | 100 | 100 | 95.6 | 91 | 100 | 91 | 100 | 86.9 | 99.2 | 88.9 | 100 | 100 |
| I |  |  |  |  |  |  |  |  |  | 100 | 95.6 | 91 | 100 | 91 | 100 | 86.9 | 99.2 | 88.9 | 100 | 100 |
| J |  |  |  |  |  |  |  |  |  |  | 95.6 | 91 | 100 | 91 | 100 | 86.9 | 99.2 | 88.9 | 100 | 100 |
| K |  |  |  |  |  |  |  |  |  |  |  | 91 | 95.6 | 90 | 95.6 | 86.9 | 96.5 | 88.9 | 95.6 | 95.6 |
| L |  |  |  |  |  |  |  |  |  |  |  |  | 91 | 96.6 | 91 | 89.9 | 99.2 | 94.7 | 91 | 91 |
| M |  |  |  |  |  |  |  |  |  |  |  |  |  | 91 | 100 | 86.9 | 99.2 | 88.9 | 100 | 100 |
| N |  |  |  |  |  |  |  |  |  |  |  |  |  |  | 91 | 87.9 | 99.2 | 91.9 | 91 | 91 |
| O |  |  |  |  |  |  |  |  |  |  |  |  |  |  |  | 86.9 | 99.2 | 88.9 | 100 | 100 |
| P |  |  |  |  |  |  |  |  |  |  |  |  |  |  |  |  | 87.9 | 90.0 | 86.9 | 86.9 |
| Q |  |  |  |  |  |  |  |  |  |  |  |  |  |  |  |  |  | 89.9 | 99.2 | 99.2 |
| R |  |  |  |  |  |  |  |  |  |  |  |  |  |  |  |  |  |  | 88.9 | 88.9 |
| S |  |  |  |  |  |  |  |  |  |  |  |  |  |  |  |  |  |  |  | 100 |
| T |  |  |  |  |  |  |  |  |  |  |  |  |  |  |  |  |  |  |  |  |

Here, A (*Escherichia coli* reference), B (*Escherichia sp*. 26N), C (*Escherichia sp*. 28N), D (*Escherichia sp*. CR1), E (*Escherichia sp*. CR2), F (*Escherichia sp*. CR4), G (*Escherichia sp*. E23), H (*Escherichia sp*. E34), I (*Escherichia sp*.G4), J (*Escherichia sp*. MCX14), K (*Escherichia sp*. NCX9), L (*Enterobacter cloacae* reference), M (*Enterobacter sp.* MCX5), N (*Enterobacter sp.* NCX14), O (*Enterobacter sp.* MCX6), P (*Klebsiella pneumoniae* reference), Q (*Klebsiella sp. MCX10*), R (*Salmonella enterica*reference), S (*Salmonella sp.* 74) and T (*Salmonella sp.* 77).

Bold numbers represent the high pairwise similarity between *Escherichia coli* reference and test isolates.


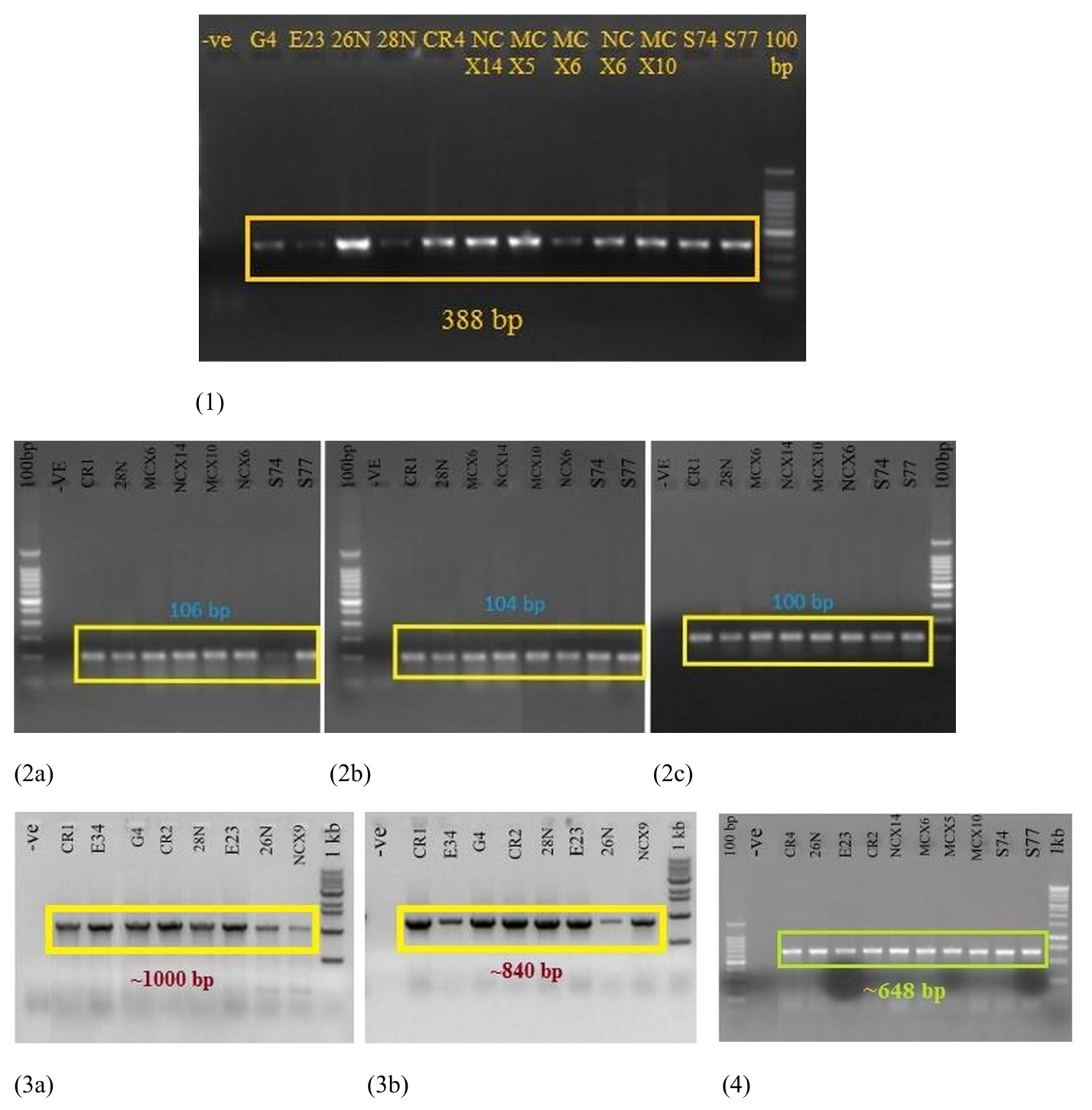


Figure S1: Image of agarose gel electrophoresis for resolving PCR amplicons of different genes from designated isolates.

1. PCR amplicons of part of *qnr*S. The molecular size marker used was 100 bp DNA Ladder (Promega, USA).

(2a), (2b) and (2c) are PCR amplicons of part of *acr*A, *acr*B and *tol*C genes respectively. The molecular size marker used was 100 bp DNA Ladder (Promega, USA).

(3a) and (3b) are PCR amplicons of *acr*R and *mar*R genes respectively. The molecular size marker used was 1kb DNA Ladder (Promega, USA).

(4) represents the PCR amplicons of part of *gyr*A. The molecular size marker used was 1kb DNA Ladder (Promega, USA).

References:

Adnan, N., Sultana, M., Islam, O. K., Nandi, S. P., & Hossain, M. A. (2013). Characterization of Ciprofloxacin resistant Extended Spectrum β-Lactamase (ESBL) producing *Escherichia* spp. from clinical waste water in Bangladesh.

Bouchakour, M., Zerouali, K., Claude, J. D. P. G., Amarouch, H., El Mdaghri, N., Courvalin, P., & Timinouni, M. (2010). Plasmid-mediated quinolone resistance in expanded spectrum beta lactamase producing *enterobacteriaceae* in Morocco. *The Journal of Infection in Developing Countries, 4*(12), 779-803.

Lane, D. (1991). 16S/23S rRNA sequencing. *Nucleic acid techniques in bacterial systematics*, 125-175.

Lindgren, P. K., Karlsson, Å., & Hughes, D. (2003). Mutation rate and evolution of fluoroquinolone resistance in *Escherichia coli* isolates from patients with urinary tract infections. *Antimicrobial Agents and Chemotherapy, 47*(10), 3222-3232.

Martínez-Martínez, L., Pascual, A., García, I., Tran, J., & Jacoby, G. A. (2003). Interaction of plasmid and host quinolone resistance. *Journal of Antimicrobial Chemotherapy, 51*(4), 1037-1039.

Nandi, S. P., Sultana, M., & Hossain, M. A. (2013). Prevalence and characterization of multidrug-resistant zoonotic *Enterobacter* spp. in poultry of Bangladesh. *Foodborne Pathogens and Disease, 10*(5), 420-427.

Sultana, M., Bilkis, R., Diba, F., & A. Hossain, M. (2014). Predominance of Multidrug Resistant Zoonotic *Salmonella* Enteritidis Genotypes in Poultry of Bangladesh. *The Journal of Poultry Science, 51*(4), 424-434.

Swick, M. C., Morgan-Linnell, S. K., Carlson, K. M., & Zechiedrich, L. (2011). Expression of multidrug efflux pump genes acrAB-tolC, mdfA, and norE in *Escherichia coli* clinical isolates as a function of fluoroquinolone and multidrug resistance. *Antimicrobial agents and chemotherapy, 55*(2), 921-924.
